# Vitamin D Deficiency Activates Gdnf-Ret-pErk1/2 Signal and Induces Kidney Malformations in Mice

**DOI:** 10.3390/ijms27073042

**Published:** 2026-03-27

**Authors:** Minghui Yu, Ningli Ye, Haixin Ju, Qianfan Miao, Chunyan Wang, Rufeng Dai, Jing Chen, Yihui Zhai, Lei Sun, Xiaohui Wu, Hong Xu, Qian Shen

**Affiliations:** 1 Department of Nephrology, Children’s Hospital of Fudan University, Shanghai Kidney Development and Pediatric Kidney Disease Research Center, National Children′s Medical Center, Shanghai 201102, China; 16211240027@fudan.edu.cn (M.Y.); 23211240022@m.fudan.edu.cn (N.Y.); hxju16@126.com (H.J.); 06301010247@fudan.edu.cn (Q.M.); chunyanxg@163.com (C.W.); dairufeng2012@163.com (R.D.); cjcjcjs@163.com (J.C.); marinezyh@163.com (Y.Z.); 2 State Key Laboratory of Genetic Engineering and National Center for International Research of Development and Disease, Institute of Developmental Biology and Molecular Medicine, Collaborative Innovation Center of Genetics and Development, School of Life Sciences, Fudan University, Shanghai 200032, China; lei_sun@fudan.edu.cn; 3 School of Basic Medical Sciences, Shanghai University of Medicine and Health Sciences, Shanghai 201318, China; xiaohui_wu@fudan.edu.cn; 4 Laboratory Animal Center, Fudan University, Shanghai 200032, China; 5 National Key Laboratory of Kidney Diseases, Beijing 100853, China

**Keywords:** congenital anomalies of the kidney and urinary tract (CAKUT), maternal vitamin D deficiency, Gdnf-Ret-p-Erk1/2

## Abstract

Congenital anomalies of the kidney and urinary tract (CAKUT) constitute the most common underlying cause of chronic kidney disease in pediatric populations. Maternal hypovitaminosis D links to mesoderm-related birth defects, leading to our hypothesis that maternal vitamin D deficiency (VDD) impairs renal development (a mesoderm-derived process) and induces offspring CAKUT. To investigate whether a low-vitamin D level can cause CAKUT, we used vitamin D-free diets to induce a maternal vitamin D deficiency mice model. The maternal vitamin D deficiency (VDD) mice models and normal vitamin D status (CON) were successfully established by administering a vitamin D-free or vitamin D-sufficient diet for 4 weeks prior to pregnancy. The overall incidence of CAKUT was significantly increased in VDD neonatal mice (19.4% vs. 2.44%; *p* = 0.0006), with a higher incidence of early duplicated budding in E11.5. E11.5 ureteric bud tissue revealed significantly increased activity of Gdnf-Ret-p-Erk1/2 signaling in the VDD group. In vivo intervention with the p-Erk1/2 antagonist U0126 in the pregnant VDD mice model at E10.5 improved CAKUT occurrence in offspring with p-Erk1/2 expression decreasing toward normal levels. Early metanephric ureteric bud H3K4me3 CUT&TAG analysis at E12.5 revealed chromatin activation patterns, which revealed that the downregulation of Hnf1β promoter region peaks was accompanied by reduced Hnf1β expression, and Robo2 promoter region peak was upregulated with increased Robo2 expression in the VDD group. Maternal vitamin D deficiency in mice significantly increased offspring CAKUT incidence. This phenotype was mediated by enhanced Gdnf-Ret-p-Erk1/2 signaling and reversed by p-Erk1/2 inhibition, with VDD inducing epigenetic remodeling of Hnf1β and Robo2 promoters.

## 1. Introduction

Congenital anomalies of the kidney and urinary tract (CAKUT) are caused by abnormal spatial and temporal regulation during kidney and urogenital tract morphogenesis. This is a broad category encompassing various developmental abnormalities of the kidneys and urogenital tract, with an incidence of approximately 3–9 per 1000 live births. It accounts for approximately 40–50% of chronic kidney disease cases that occur before age 30 [1,2,3]. Approximately one-third of CAKUT patients may also present with abnormalities in extrarenal structures [4,5].

CAKUT exhibits significant clinical variability and complex etiology. To date, only 50 monogenic variants have been identified as causative mutations in human CAKUT, accounting for approximately 6–20% of cases [6,7]. A substantial proportion of CAKUT cases remain unexplained. Therefore, most scholars believe that genetic factors, environmental factors, and interactions between genes and the environment can lead to the occurrence of CAKUT. Studies have indicated that maternal exposure to adverse environmental factors before and during pregnancy elevates the risk of CAKUT in offspring. Large-scale case–control studies have revealed associations between maternal obesity, diabetes, chronic kidney disease, acute infections during pregnancy, and medication use with elevated CAKUT risk in offspring [8,9].

Vitamins are known to regulate renal development. Previous studies and our own research have already demonstrated that maternal vitamin A deficiency increases the risk of CAKUT [10,11]. Previous studies have demonstrated an association between maternal hypovitaminosis D and adverse pregnancy outcomes in human populations [12,13]. Some evidence suggests that maternal hypovitaminosis D may be linked to mesoderm-related birth defects such as lower urinary tract malformations and circulatory system abnormalities [14,15]. Thus, we hypothesized the kidney as the mesoderm-related organ that might be disrupted by maternal vitamin D deficiency, resulting in CAKUT in offspring.

This study aimed to establish prepregnancy and prepregnancy + gestational vitamin D deficiency mouse models to investigate the effects of maternal vitamin D deficiency on offspring kidney development. It will also investigate whether adequate vitamin D supplementation during pregnancy can reduce the incidence of CAKUT in offspring, improve their long-term vitamin D status, and elucidate the potential underlying mechanisms.

## 2. Results

### 2.1. Successful Establishment of Vitamin D-Deficient Maternal Mouse Models

**Figure 1:**
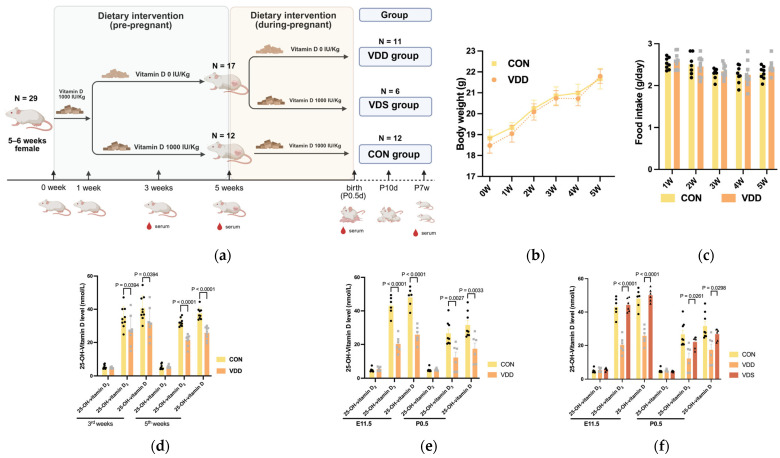
Prepregnant plus pregnant vitamin D-deficient and pregnancy vitamin D-deficient mice model. (**a**) Schematic diagram of modeling of vitamin D-deficient mice; (**b**) Body weight among 2 groups during modeling; (**c**) Daily food intake among 2 groups during modeling; (**d**) vitamin D levels (nmol/L) at 3rd and 5th weeks of modeling; (**e**,**f**) vitamin D levels (nmol/L) at E11.5d and P0.5d of modeling. Data is shown as the mean ± SEM. Abbreviations: VDD, pre-pregnancy plus pregnancy vitamin D-deficient group; VDS, pre-pregnancy vitamin D-deficient group; CON, pre-pregnancy plus pregnancy normal group.

Maternal vitamin D deficiency mouse models and normal vitamin D status (CON) were successfully established by administering a vitamin D-free diet or a normal vitamin D diet for 4 weeks prior to pregnancy. During modeling, maternal body weight and food intake were measured weekly and did not significantly differ between the groups (Figure 1b,c). LC–MS analysis of serum 25-hydroxyvitamin D levels at 3 and 5 weeks after modeling revealed significantly lower concentrations in vitamin D-deficient maternal mice than in control mice at week 5 (25.77 ± 3.70 vs. 37.46 ± 3.48 nmol/L, *p* < 0.0001) (Figure 1d). No statistically significant difference was observed in calcium and phosphorus concentrations between the two groups (Supplement Figure S1A,B).

**Figure 2:**
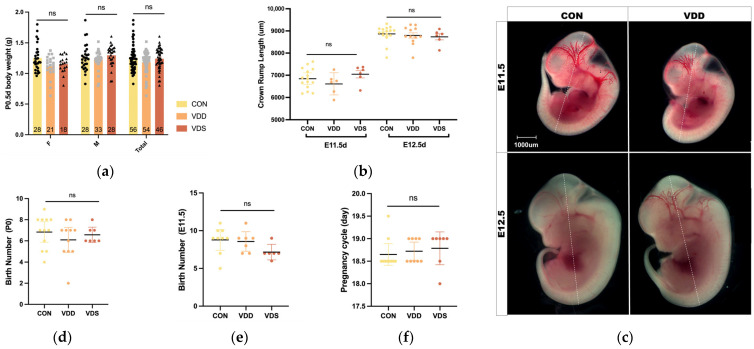
Pregnancy and delivery in model mice. (**a**) Body weights of the newborn mice; (**b**) Length of top rump of the embryos at E11.5 or E12.5; (**c**) Gestation cycle; (**d**,**e**) Number of offspring in the neonatal and early embryonic phases; (**f**) Gestation cycle. Data is shown as the mean ± SEM; Abbreviations: ns, non-significant; VDD, pre-pregnancy plus pregnancy vitamin D-deficient group; VDS, pre-pregnancy vitamin D-deficient group; CON, pre-pregnancy plus pregnancy normal group; F: Female; M: Male.

No significant differences were observed in the body weights of newborn mice among the groups (Figure 2a). The crown-rump lengths of embryos at E11.5 and E12.5 did not differ across groups (Figure 2b,c). Intergroup comparisons revealed no differences in the number of neonatal offspring or early-stage embryos (Figure 2d,e). Additionally, the gestation periods of the dams showed no differences across groups (Figure 2f).

LC–MS analysis of serum 25-hydroxyvitamin D levels in maternal mice at E11.5 and P0.5 revealed significantly lower concentrations in the E11.5 VDD group than in the CON group (25.81 ± 5.10 vs. 48.07 ± 5.67 nmol/L, *p* < 0.0001). The serum 25-hydroxyvitamin D levels in the P0.5 VDD group also significantly decreased compared with those in the CON group (17.43 ± 8.28 vs. 31.61 ± 7.78 nmol/L, *p* = 0.0033), with both groups exhibiting serum 25-hydroxyvitamin D levels below normal ranges (Figure 1e). Compared with the control group, the VDS group, which received normal vitamin D supplementation during pregnancy, had significantly elevated serum 25-hydroxyvitamin D levels (E11.5 days: 55.11 ± 3.82 vs. 25.81 ± 5.10 nmol/L, *p* < 0.0001; P0.5 days: 26.75 ± 3.52 vs. 17.43 ± 8.28 nmol/L, *p* = 0.0298) (Figure 1f).

### 2.2. Maternal Vitamin D-Deficiency Increased Offspring CAKUT Incidence

**Figure 3:**
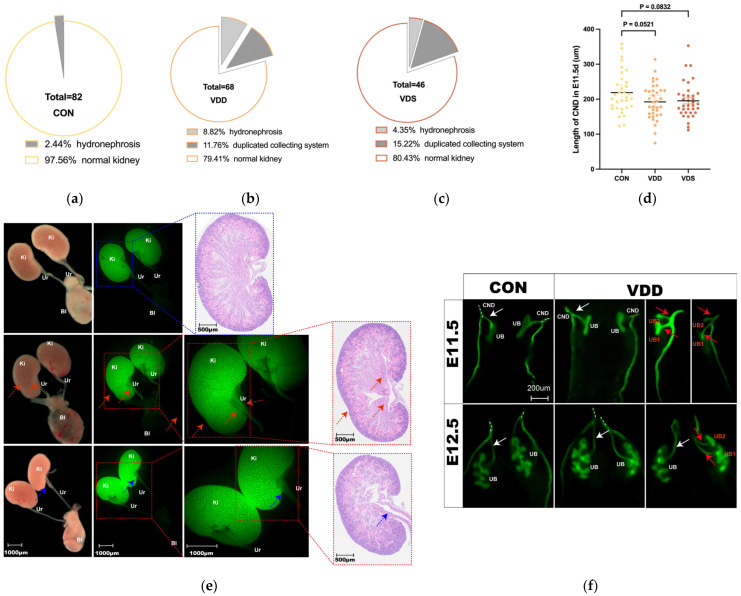
Maternal vitamin D-deficient mice cause offspring CAKUT. (**a**–**c**) Incidence and phenotype of CAKUT among 3 groups; (**d**) CND length; (**e**) phenotype of CAKUT in VDD group (visualized by Hoxb7-GFP expression); blue dashed box represent normal kidney microscopic and HE images; red and blue arrow, red dashed boxes represent abnormal kidney microscopic and HE images; (**f**) abnormal diagram of ureteric bud sprouting at E11.5; white arrow and white dashed line for normal UB and CND, while red arrow and red dashed line for ectopic UBs (UB1, UB2) and abnormal CND; data is shown as the mean ± SEM. Abbreviations: VDD, pre-pregnancy plus pregnancy vitamin D-deficient group; VDS, pre-pregnancy vitamin D-deficient group; CON, pre-pregnancy plus pregnancy normal group; CND, common nephric duct; UB, ureteric bud; Ki, kidney; Ur, ureter; Bl, bladder.

Observations of neonatal kidney phenotypes in offspring mice revealed a significantly greater incidence of CAKUT in VDD [19.40% (13/67, N = 11) vs. 2.44% (2/82, N = 12), *p* < 0.0001, Power = 95.38%] (Figure 3a,b). To account for potential litter effects in the animal experiments, the CAKUT incidence was calculated at the litter level and analyzed using GEE clustered by litter. The CAKUT incidence in the VDD group was also higher than the CON group (*p* = 0.001), RR = 2.26 (95% CI: 0.90–3.62). Among the offspring, the incidence of CAKUT was slightly greater in males than in females [24.40% (10/41) vs. 11.54% (3/26), *p* = 0.3714]. The CAKUT phenotypes of the VDD offspring were as follows: There were 11.94% (8/67) duplicated collecting systems and 7.46% (5/67) hydronephrosis, with detailed phenotype diagrams shown in Figure 3e. Prenatal vitamin D supplementation improved maternal vitamin D levels but did not reduce the incidence of CAKUT among offspring. The CAKUT incidence in the VDS group was similar to that in the VDD group at 19.57% (9/46, N = 6) and was higher than that in the normal group (Figure 3c) (*p* = 0.004), RR = 2.26 (95% CI: 0.74–3.78), Power = 89.80%. The distribution of CAKUT phenotypes in the VDS offspring was comparable to that in the VDD group.

No significant intergroup differences were observed in body weight, kidney weight, kidney length, or kidney-to-body weight ratio among newborn mice and P10.5 mice, and no sex-specific differences were detected either (Supplemental Figure S1C–I). At 7 postnatal weeks, offspring kidney weight, kidney length, and kidney weight/body weight ratio showed no significant variation across groups; however, male offspring generally exhibited higher kidney weight and kidney weight/body weight ratio than female offspring (Supplemental Figure S1J–L). Further LC–MS analysis of vitamin D levels at 7 weeks revealed no intergroup differences, with no sex-related variations noted (Supplemental Figure S1M).

### 2.3. Maternal Vitamin D-Deficiency Induced Embryonic Ureteric Bud Abnormalities

Observations of ureteric bud emergence at E11.5 and E12.5 revealed that the duplicated emergence rates in the VDD and VDS groups were higher than the control group, 7.41% (6/81), 6.78% (4/59), and 2.54% (1/65), respectively (Figure 3d). Measurements of the length of the common nephric duct (CND) at E11.5 revealed a trend toward shortening in the VDD and VDS groups compared with the control group (192.30 ± 8.58 vs. 195.30 ± 8.61 vs. 218.90 ± 10.39 μm, p = 0.0521, p = 0.0832) (Figure 3e).

### 2.4. Maternal Vitamin D-Deficiency Activated Gdnf/Ret/p-Erk1/2 During Metanephric Development

**Figure 4:**
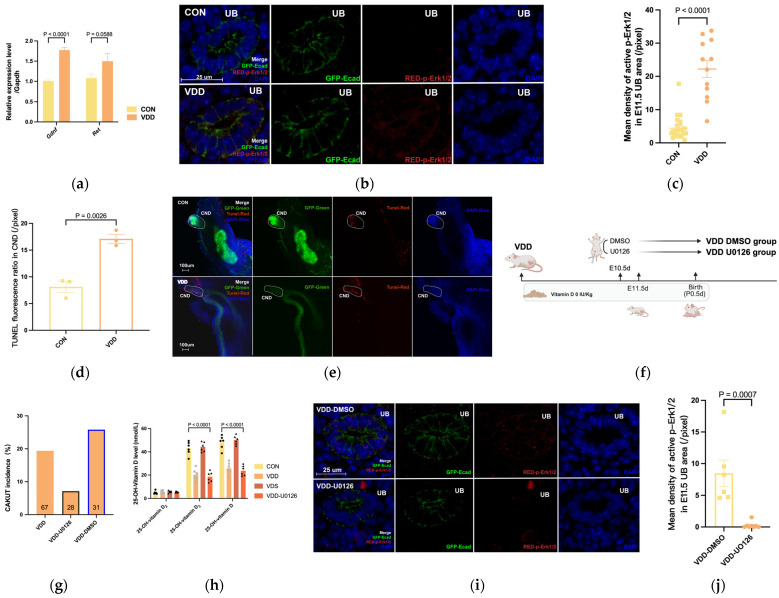
Detect the changes in Gdnf/Ret/p-Erk1/2 and rescue. (**a**) Detect the Gdnf/Ret expression levels of UB tissues by qRT–PCR; (**b**,**c**) Detect the p-Erk1/2 expression levels of UB tissues by immunofluorescence; (**d**,**e**) Detect apoptosis by Tunel detection kit of CND; (**f**,**g**) Schematic diagram of U0126 rescue model for a single intraperitoneal injection in E10.5d and CAKUT incidences; (**h**) vitamin D levels (nmol/L) at E11.5d of modeling; (**i**,**j**) Detect the p-Erk1/2 expression levels of UB tissues by immunofluorescence after Administration of U0126. Each value is expressed as the mean ± SEM. Abbreviations: VDD, pre-pregnancy plus pregnancy vitamin D-deficient group; VDS, pre-pregnancy vitamin D-deficient group; CON, pre-pregnancy plus pregnancy normal group; UB, Ureteric Bud; CND, common nephric duct; DMSO, placebo; U0126, p-ERK1/2 antagonist.

qRT-PCR analysis of the expression of the core kidney development pathway Gdnf/Ret expression at E11.5 was upregulated in the VDD group as shown in Figure 4a. Immunofluorescence staining revealed molecular changes in p-Akt/p-Plcγ/p-Erk1/2—key downstream phosphorylated molecules of Ret—in E11.5 ureteric buds across all groups, indicating elevated p-Erk1/2 expression in VDD tissues compared with controls (Figure 4b,c). p-Plcγ expression was reduced compared with that in the control group (Supplemental Figure S2A,B), whereas p-Akt expression was not significantly different between the two groups (Supplemental Figure S2C,D). Increased apoptosis was observed in the CND of VDD embryos at E11.5 by immunofluorescent TUNEL staining (Figure 4d,e). Following intervention with the p-Erk1/2 inhibitor U0126 at gestational day E10.5, the incidence of CAKUT in newborn mice decreased in the VDD group (Figure 4f,g). Additionally, the administration of U0126 failed to correct the vitamin D-deficient state in VDD (Figure 4h), but instead reduced the p-Erk1/2 expression in UB tissue at E11.5 (Figure 4i,j).

### 2.5. H3K4me3 Modification Profiles and Functional Analysis of Differentially Enriched Genes in E12.5 Embryonic Kidneys

**Figure 5:**
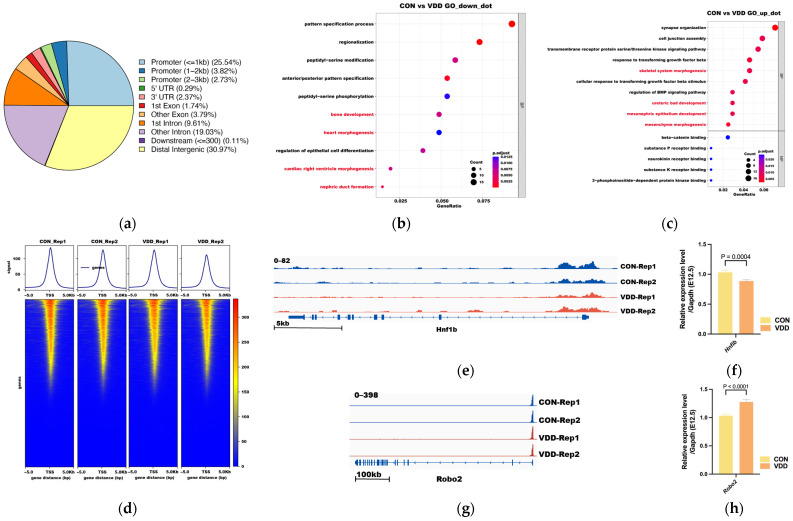
H3K4me3 Modification Profiles and Functional Analysis of Differentially Enriched Genes in E12.5 Embryonic Kidneys. (**a**) Distribution of CUT&TAG peaks of H3K4me3 across genomic regions in 2 groups; (**b**) GO Class of Differentially Downregulated Molecules in E12.5 UB tissues; (**c**) GO Class of Differentially Upregulated Molecules in E12.5 UB tissues; (**d**) Average read density across transcription start sites (TSS) and chromatin occupancy heatmaps of H3K4me3 for 2 groups; (**e**–**h**) H3K4me3 CUT&TAG Hnf1β and Robo2 promoter region peak and detect the expression levels by qRT–PCR; results are shown for the two biological replicates; Data is shown as the mean ± SEM. Abbreviations: TSS, transcription start sites; VDD, pre-pregnancy plus pregnancy vitamin D-deficient group; CON, pre-pregnancy plus pregnancy normal group.

H3K4me3 CUT&TAG analysis was performed to examine chromatin promoter activation in E12.5 embryonic kidney tissues from the VDD and CON groups. Enriched genes in both groups were located near transcription start sites (TSSs), with differentially enriched genes predominantly concentrated in promoter regions (22.91%) (Figure 5a,d). GO enrichment analysis of differentially expressed genes (DEGs) revealed significant enrichment in development-related pathways, including those associated with the development of organs such as the ureteric bud, heart, and skeleton (Figure 5b,c, Supplementary Tables S2 and S3).

Analysis of promoter region peaks around the Gdnf/Ret axis—a key signaling pathway in kidney development—and qT–PCR validation revealed that downregulation of the Hnf1β promoter region peak was accompanied by decreased Hnf1β expression in the VDD group (Figure 5e,f) and that upregulation of the Robo2 promoter region peak was accompanied by increased Robo2 expression (Figure 5g,h); other molecules, such as Ret and Foxc1, showed downregulated promoter region peaks, whereas Bmp4 and Gata3 showed upregulated promoter region peaks. However, no corresponding changes in transcription were observed (Supplemental Figure S3).

## 3. Discussion

A vitamin D-free diet resulted in the successful establishment of a maternal vitamin D deficiency mouse model. Offspring from the maternal vitamin D deficiency group exhibited increased rates of neonatal urinary tract developmental abnormalities, accompanied by embryonic ureteric bud abnormalities. Enhanced Gdnf-Ret-p-Erk1/2 signaling in VDD embryonic ureteric buds mediated this phenotype, which was reversed by p-Erk1/2 inhibition. Epigenetic analysis further identified the VDD-driven chromatin remodeling of Hnf1β and Robo2 promoters.

Population studies [16] suggest that reduced dietary vitamin D intake may be associated with selective congenital anomalies, particularly musculoskeletal defects. Compared with high preconceptional dietary vitamin D intake (>107.55 IU/day), low intake (<65.21 IU/day) was linked to significantly increased risks of anencephaly (aOR = 1.28, 95% CI 1.01–1.63), hypospadias (aOR = 1.21, 95% CI 1.04–1.40), ventricular septal defects (aOR = 1.16, 95% CI 1.05–1.30), diaphragmatic hernia (aOR = 1.42, 95% CI 1.13–1.79), and abdominal wall defects (aOR = 1.27, 95% CI 1.07–1.52). However, this study did not measure maternal vitamin D levels [14]. Koster MPH conducted a case–control study involving 245 mothers of children with CHD and 432 mothers of children without CHD at four tertiary hospitals in the Netherlands between 2003 and 2005 (defined vitamin D deficiency as a concentration <75 nmol/L [17]). They reported that vitamin D deficiency was associated with CHD in offspring (OR = 2.15, 95% CI 1.44–3.19) [18]. However, the effect of vitamin D deficiency on kidney development remained scarce. A population study by Miliku K [19] did not report an association between maternal vitamin D levels and offspring kidney size. There is conflicting information in the literature regarding the effect of Vitamin D on kidney development. While Rogers et al. observe increased glomeruli in metanephroi treated with Vitamin D [20], Maka et al. report that Vitamin D deficiency stimulates nephrogenesis in the rat [21]. Neither of these studies detected the ureter duplication or hydronephrosis. In contrast, the current study, conducted at the animal mice model level, clearly demonstrated that prepregnancy vitamin D deficiency can increase the risk of urinary tract developmental abnormalities in offspring to a certain extent.

Owing to dietary patterns, sunlight exposure, and geographical factors, vitamin D deficiency or insufficiency is highly prevalent in the general population [22]. Pregnant women are particularly susceptible to vitamin D deficiency [23,24]. The prevalence of vitamin D deficiency among pregnant women in China is approximately 41.96% [25], with an even higher rate of 65.26% reported in the eastern coastal regions of the country. Kozeta Miliku et al. reported a 47.6% prevalence of vitamin D deficiency (defined as a serum 25-hydroxyvitamin D concentration <50 nmol/L) in a cohort of 4212 mother–child pairs in the Netherlands [26]. Vitamin D3 is a lipophilic molecule, with 25-hydroxyvitamin D3 [25(OH)D3] being its primary metabolite and the predominant storage form in blood (approximately 80%) [27]. Metabolic changes in 25-hydroxyvitamin D [25(OH)D] occur during pregnancy, characterized by increased vitamin D requirements in early gestation, with metabolite levels at 12 weeks gestation being three times greater than those in nonpregnant women [28]. Our study also revealed that at E11.5, maternal 25(OH)D levels in the control group approximately doubled compared with prepregnancy levels, whereas postpartum 25(OH)D levels decreased significantly, mirroring the pattern seen in pregnant human women. Thus, under vitamin D-deficient diet conditions, the vitamin D levels of the VDD group at E11.5 failed to meet the requirements of pregnancy, potentially contributing to urinary system developmental abnormalities in offspring. Previous population studies suggest that vitamin D supplementation during pregnancy can increase maternal vitamin D levels. This study also attempted normal vitamin D supplementation during pregnancy, which indeed increased maternal vitamin D levels to control levels. However, despite elevated vitamin D levels, the offspring CAKUT incidence did not improve. These findings thus imply that earlier assessment and monitoring of vitamin D status in women of childbearing age during the periconceptional period are warranted. Meanwhile this finding also indicates that alterations downstream of vitamin D’s biological actions may not have been corrected. Multiple studies have indicated that GDNF/RET is a key signaling pathway in metanephric kidney development [29]. AKT/PI3K, RAS-ERK1/2/MAPK, and PLCγ/Ca^++^ represent critical downstream pathways activated by RET that mediate cellular functions [29,30]. This study also validated the activation of the key downstream signal p-Erk1/2 via qRT-PCR and immunofluorescence. Attempts to apply the p-Erk1/2 inhibitor U0126 significantly reduced the incidence of CAKUT in newborn mice from the VDD group and decreased p-Erk1/2 expression in the UB tissues without correcting the vitamin D-deficient state in VDD.

Research on the physiological effects of vitamin D and its metabolites on the fetus is limited [31]. Precise regulation of development-related genes is critically important during embryogenesis and cardiac development, particularly between gestational weeks 2 and 7 [32]. Vitamin D is synthesized into its active form, 1,25(OH)_2_D_3_, in the body via the skin, liver, and kidneys. It exerts genomic and nongenomic regulatory effects by binding to the vitamin D receptor (VDR) [32]. Studies have also revealed that vitamin D and VDR have diverse effects on the epigenome [32]. The ligand-dependent histone modifications H3K4me3 and H3K27ac can be used to distinguish the regulatory roles of specific transcription factors at promoters and enhancers, respectively. Veijo Nurminen utilized chromatin immunoprecipitation sequencing (ChIP-seq) datasets and machine learning to classify 1,25(OH)_2_D_3_-sensitive histone modifications, identifying 260 regions bearing H3K4me3 modifications and 287 regions bearing H3K27ac modifications. These sites reside within the promoter regions and associated enhancers of 59 VDR target genes, confirming the epigenetic effects of 1,25(OH)_2_D_3_ [33,34]. In this study, we first observed that maternal vitamin D deficiency significantly disrupted the expression of key molecules involved in metanephric development in ureteric bud tissue. Furthermore, using the CUT&TAG method, we found that maternal vitamin D deficiency interferes with H3K4me3 enrichment in the promoter regions of these key metanephric development molecules such as Ret, hnf1β, Robo2. This finding provides partial epigenetic evidence explaining the association between vitamin D deficiency and adverse urinary system outcomes.

The limitations of this study include the detection of vitamin D concentration solely in maternal serum, without the direct measurement of vitamin D levels in embryos or developing kidneys, thus failing to directly reflect fetal vitamin D status. Furthermore, this research lacks support from large-scale population data and is confined to mouse models.

## 4. Materials and Methods

### 4.1. Establishment of Vitamin D-Deficient Mouse Models

In this study, FVB/NJ mice were crossed with Hoxb7-mVnus fluorescent mice, enabling green fluorescent labeling of urinary tract epithelial tissues. Experimental mice were housed in an SPF-grade animal facility at the Laboratory Animal Center Fudan University, and handled in accordance with the Institute’s Animal Care and Use Committee regulations on experimental animal welfare and management. Ethics approval number: IDM2022032.

A mouse model was established using a specialized dietary regimen (feed supplied by Nantong Trophic Feed Technology Co., Ltd., Nantong, China). Diets were categorized based on vitamin D content: A vitamin D-free diet (0 IU/kg) and a vitamin D-sufficient diet (1000 IU/kg), with all other nutritional components held constant. The modeling methodology followed that, reported by Belenchia, A.M. [15]. Female mice aged 5–6 weeks were randomly allocated to either the vitamin D-deficient group or the control group via a random number table method. The vitamin D-deficient group was fed a vitamin D-sufficient diet for 1 week followed by a vitamin D-free diet for 4 weeks, whereas the control group received the same vitamin D-sufficient diet continuously for 5 weeks. The mice were housed in groups at a female-to-male ratio of 3–4:1. Pregnancy status in females was assessed twice daily. An embryonic day 0.5 (E0.5) was defined as the earliest observation of a vaginal plug. Following the detection of the vaginal plug, the vitamin D-deficient group was randomly assigned to either the prepregnancy + gestational vitamin D deficiency group (VDD) (receiving a vitamin D-free diet throughout gestation) (n = 11) or the prepregnancy vitamin D deficiency + gestational vitamin D supplementation group (VDS) (receiving a normal vitamin D-sufficient diet throughout gestation) (n = 6). The control group (CON) (n = 12) was fed a consistent vitamin D-sufficient diet throughout gestation until spontaneous delivery. All pregnant mice across the three groups had free access to food and water (experimental design illustrated in Figure 1a). Starting from E16.5, observations were conducted twice daily to detect pup delivery, with the earliest observed birth time designated as postnatal day 0.5 (P0.5). Postpartum, all pregnant mice in all groups were fed a normal vitamin D-containing diet until the pups reached 7 weeks of age.

### 4.2. Serum Vitamin D and Calcium

Serum samples were collected at approximately 9 a.m. from model mice fed the specialized diet at 3 weeks, 5 weeks, embryonic day 11.5 (E11.5), and parturition, as well as from offspring at 7 weeks of age. Serum levels of multiple vitamin D metabolites, including 25-hydroxyvitamin D2 Standards (CDAA-741041, Anpel, Shanghai, China) and 25-hydroxyvitamin D3 Standards (CDAA-741042, Anpel, Shanghai, China) via ACQUITY UPLC H-class/Xevo TQ-XS (Waters, Milford, MA, USA). Serum 25-hydroxyvitamin D levels were calculated as the sum of the 25-hydroxyvitamin D2 and 25-hydroxyvitamin D3 levels. Serum calcium and phosphate were measured using a Calcium Assay kit (ab102505, Abcam, Shanghai, China) or Phosphate Assay kit (ab65622, Abcam, Shanghai, China).

### 4.3. Phenotypic Analysis

Gross phenotypic characteristics, including the quantity and morphology of the kidneys, ureters, and bladders, were observed under a fluorescence microscope in three groups of newborn mice. Pregnant mice at embryonic day (E) 11.5 and E12.5 were anesthetized with carbon dioxide (CO_2_), after which embryonic kidneys were isolated and examined under a fluorescence stereomicroscope to assess the number, position, and branching pattern of ureteric buds.

### 4.4. qRT–PCR

Embryonic kidneys from three groups were isolated at E11.5, and total RNA was extracted using an RNeasy Mini Kit (74104, Qiagen, Hilden, Germany). Total RNA was isolated from the three groups at E12.5 days using the TRIzol method. Equal amounts (500–2000 ng) of RNA were reverse transcribed into cDNA. Quantitative Real-time PCR (qRT-PCR) amplification and analysis were performed, with three parallel reaction tubes per group and three samples per group, and the samples were added in the dark. The primers used were shown in Supplementary Table S1.

### 4.5. Immunofluorescence Staining of Embryonic Kidney Tissue

E11.5 embryos were dissected under a stereomicroscope, fixed with formaldehyde, paraffin-embedded, and sectioned. Sections were stained with anti-E-cadherin antibody (GB12082; Servicebio, Wuhan, China; 1:500), p-Akt antibody (9271; Cell Signaling Technology, Boston, MA, USA; 1:200), p-Erk1/2 antibody (4370S; Cell Signaling Technology, Boston, MA, USA; 1:200), and p-PLCγ antibody (8713; Cell Signaling Technology, Boston, MA, USA; 1:200). Nuclei were stained with DAPI (G1012; Servicebio, Wuhan, China).

### 4.6. Detection of Apoptosis in Embryonic Kidney Tissue

Embryos at E11.5 were dissected under a stereomicroscope, fixed with formaldehyde, paraffin-embedded, and sectioned. Apoptosis markers were detected using a one-step TUNEL apoptosis detection kit (C1089; Beyotime, Shanghai, China).

### 4.7. H3K4me3 Cut-Tag

E12.5 embryonic kidney tissue was extracted and dissociated into a single-cell suspension. After passing the cell counting and viability tests, libraries were constructed using the Vazyme (TD904) kit (Vazyme, Nanjing, China) protocol. The samples were then sent to Novogene (Novogene Co., Ltd., Beijing, China) for quality control, sequencing, and analysis.

### 4.8. Data Analysis

All statistical analyses in this study were performed using GraphPad Prism 10 and SPSS (20.0.0) software. Results are expressed as mean ± standard deviation (SD). A *p* < 0.05 was considered statistically significant, whereas *p* > 0.05 indicated no statistical significance (ns). Categorical data were analyzed by the chi-square test, and intergroup comparisons were conducted using two-tailed unpaired *t*-tests. To account for the non-independence of offspring nested within litters (clustering effect) and eliminate litter-based pseudo-replication, a Generalized Estimating Equation (GEE) model with a logit link function was employed for analyzing the binary outcome (CAKUT occurrence). A priori power analysis was conducted to determine the minimum number of litters required per group to detect a meaningful difference in the incidence of CAKUT. Based on preliminary experiments, CAKUT incidence in the VDD group was assumed to be 19%. With a significance level of 0.05, a desired statistical power of 90% and the average number of pups per litter of 6–8, calculations indicated that at least 8–12 litters per group would be needed.

## 5. Conclusions

In summary, this study found maternal vitamin D deficiency can cause abnormal metanephric development in offspring mice, manifested as increased duplication of ureteric bud outgrowths, leading to significantly increased CAKUT incidence. Enhanced Gdnf-Ret-p-Erk1/2 signaling in VDD embryonic ureteric buds mediated this phenotype, which was reversed by p-Erk1/2 inhibition. Epigenetic analysis further identified VDD-driven chromatin remodeling of Hnf1β and Robo2 promoters.

## Data Availability

The raw data of CUT&TAG have been uploaded to the GEO website. To review GEO accession GSE315190: Go to https://www.ncbi.nlm.nih.gov/geo/query/acc.cgi?acc=GSE315190 (accessed on 23 March 2026).

## Supplementary Materials

The following supporting information can be downloaded at: https://www.mdpi.com/article/10.3390/ijms27073042/s1.

## Abbreviations

The following abbreviations are used in this manuscript:

CAKUT	Congenital anomalies of the kidney and urinary tract
VDD	Vitamin D deficiency
CON	Normal vitamin D status
