# Supplementary material for: Vitamin D Deficiency Activates Gdnf-Ret-pErk1/2 Signal and Induces Kidney Malformations in Mice
Source: Int J Mol Sci. 2026 Mar 27;27(7):3042. doi: 10.3390/ijms27073042 (PMC13073290; doi:10.3390/ijms27073042)
Supplement: Supplementary file 1 [file ijms-27-03042-s001.zip › ijms-4158801-supplementary.pdf]

Vitamin D Deficiency Activates Gdnf-Ret-pErk1/2 Signal and Induces Kidney Malformations in Mice

Minghui Yu [1,†], Ningli Ye [1,†], Haixin Ju [1], Qianfan Miao [1], Chunyan Wang [1], Rufeng Dai [1], Jing Chen [1], Yihui Zhai [1], Lei Sun [2], Xiaohui Wu [3,4], Hong Xu [1,5,*] and Qian Shen [1,5,*]

1 Department of Nephrology, Children's Hospital of Fudan University, Shanghai Kidney Development and Pediatric Kidney Disease Research Center, National Children's Medical Center, Shanghai 201102, China; 16211240027@fudan.edu.cn (M.Y.); 23211240022@m.fudan.edu.cn (N.Y.); hxju16@126.com (H.J.); 06301010247@fudan.edu.cn (Q.M.); chunyanxg@163.com (C.W.); dairufeng2012@163.com (R.D.); cjcjcjs@163.com (J.C.); marinezyh@163.com (Y.Z.)
2 State Key Laboratory of Genetic Engineering and National Center for International Research of Development and Disease, Institute of Developmental Biology and Molecular Medicine, Collaborative Innovation Center of Genetics and Development, School of Life Sciences, Fudan University, Shanghai 200032, China; lei_sun@fudan.edu.cn
3 School of Basic Medical Sciences, Shanghai University of Medicine and Health Sciences, Shanghai 201318, China; xiaohui_wu@fudan.edu.cn
4 Laboratory Animal Center, Fudan University, Shanghai 200032, China
5 National Key Laboratory of Kidney Diseases, Beijing, China
* Correspondence: hxu@shmu.edu.cn (H.X.); shenqian@shmu.edu.cn (Q.S.)
† These authors contributed equally to this work.

Inventory of supplementary materials

I. Supplementary Tables

Table S1. The primers used for quantitative RT-PCR

Table S2. GO enrichment analysis of differentially expressed upregulated genes by H3K4me3 CUT&TAG (TOP30)

Table S3. GO enrichment analysis of differentially expressed downregulated genes by H3K4me3 CUT&TAG (TOP30)

II. Supplementary Figures

Supplementary Figure S1. Kidney changes in offspring mice with prepregnant plus pregnant vitamin D-deficient and pregnancy vitamin D-deficient

Supplementary Figure S2. Detect the changes of p-Akt and p-Plcγ

Supplementary Figure S3. H3K4me3 CUT&TAG Ret, Foxc1, Bmp4, Gata3 promoter region peak and detect the expression levels by qRT–PCR

Table S1. The primers used for quantitative RT-PCR

Gene name	Primers
**Ret-forward**	AGTTATTCCTCCTCAGGCACCC
**Ret-reverse**	CCTTCTCCCAGAGTTTTCCCAAG
**Gdnf- forward**	TCCAGGGTATGAATCCATTGAGTG
**Gdnf-reverse**	GCAAAGAACCAAGCCAGTGTATCTC
**Robo2-forward**	TTTTTGCGAATTGTTCATGGGC
**Robo2--reverse**	ATTTCGACTCACTGCTTCACC
**Hnf1β-forward**	AGGGAGGTGGTCGATGTCA
**Hnf1β-reverse**	TCTGGACTGTCTGGTTGAACT
**Bmp4-forward**	GCAAGTTTGTTCAAGATTGGCTCC
**Bmp4-reverse**	CCATCAGCATTCGGTTACCAGG
**Gata3-forward**	CCAGGCAAGATGAGAAAGAGTG
**Gata3-reverse**	ATAGGGCGGATAGGTGGTAATG
**Foxc1-forward**	CCCCGGACAAGAAGATCACTC
**Foxc1-reverse**	AGGTTGTGCCGTATGCTGTTC
**Gapdh-forward**	TGTTCCTACCCCCAATGTGTCC
**Gapdh-reverse**	GGAGTTGCTGTTGAAGTCGCAG

Table S2. GO enrichment analysis of differentially expressed upregulated genes by H3K4me3 CUT&TAG (TOP30).

Description	GeneRatio	BgRatio	pvalue	p.adjust	qvalue	geneID	Count
synapse organization	17/239	489/29008	7.48E-07	0.0025	0.0019	Utrn/Neurod2/Mdga2/Sipa1l1/Cacnb3/Opa1/Synpo/Flrt1/Abhd17b/Lrrc4c/Nfia/Rims3/Add2/Atp2b2/Eef2k/Insr/Gpm6a	17
response to transforming growth factor beta	11/239	230/29008	3.63E-06	0.0054	0.0042	Mstn/Ptprk/Fut8/Smad2/Eng/Map3k7/Ltbp4/Dand5/Cdh5/Zfhx3/Wwox	11
**mesenchyme morphogenesis**	6/239	53/29008	4.88E-06	0.0054	0.0042	Osr1/Foxc1/Smad2/Eng/Rbpj/Dchs1	6
**skeletal system morphogenesis**	11/239	261/29008	1.20E-05	0.0071	0.0056	Osr1/Dync2i1/Sfrp4/Foxc1/Smad2/Sp5/Tmem119/Trpv4/Comp/Wwox/Sik3	11
cell junction assembly	14/239	439/29008	1.90E-05	0.0071	0.0056	Tns1/Ptprk/Lims1/Bcas3/Mdga2/Sipa1l1/Flrt1/Trpv4/Add2/Dchs1/Eef2k/Gpm6a/Cdh5/Cldn34d	14
cellular response to transforming growth factor beta stimulus	10/239	226/29008	1.98E-05	0.0071	0.0056	Mstn/Ptprk/Fut8/Smad2/Eng/Map3k7/Ltbp4/Dand5/Cdh5/Wwox	10
transmembrane receptor protein serine/threonine kinase signaling pathway	13/239	387/29008	2.20E-05	0.0071	0.0056	Mstn/Ptprk/Fut8/Sfrp4/Fstl1/Smad2/Eng/Map3k7/Rbpj/Ltbp4/Comp/Dand5/Cdh5	13
**regulation of BMP signaling pathway**	7/239	102/29008	2.24E-05	0.0071	0.0056	Sfrp4/Fstl1/Smad2/Eng/Rbpj/Dand5/Cdh5	7
**ureteric bud development**	7/239	104/29008	2.54E-05	0.0071	0.0056	Osr1/Foxc1/Epcam/Greb1l/Smad2/Pax8/Dchs1	7
**mesonephric epithelium development**	7/239	104/29008	2.54E-05	0.0071	0.0056	Osr1/Foxc1/Epcam/Greb1l/Smad2/Pax8/Dchs1	7
**mesonephric tubule development**	7/239	104/29008	2.54E-05	0.0071	0.0056	Osr1/Foxc1/Epcam/Greb1l/Smad2/Pax8/Dchs1	7
transforming growth factor beta receptor signaling pathway	9/239	186/29008	2.57E-05	0.0071	0.0056	Mstn/Ptprk/Fut8/Smad2/Eng/Map3k7/Ltbp4/Dand5/Cdh5	9
**mesonephros development**	7/239	107/29008	3.06E-05	0.0077	0.0060	Osr1/Foxc1/Epcam/Greb1l/Smad2/Pax8/Dchs1	7
**bone morphogenesis**	7/239	108/29008	3.25E-05	0.0077	0.0060	Sfrp4/Foxc1/Sp5/Tmem119/Trpv4/Comp/Sik3	7
**bone development**	10/239	243/29008	3.67E-05	0.0082	0.0064	Sfrp4/Foxc1/Sp5/Map3k7/Tmem119/Trpv4/Akap13/Dchs1/Comp/Sik3	10
substance P receptor binding	2/243	2/28438	7.27E-05	0.0237	0.0195	Tac4/Tac1	2
BMP signaling pathway	8/239	173/29008	9.93E-05	0.0207	0.0162	Sfrp4/Fstl1/Smad2/Eng/Rbpj/Comp/Dand5/Cdh5	8
positive regulation of MAP kinase activity	7/239	130/29008	1.06E-04	0.0207	0.0162	Pik3cg/Mapk8ip3/Map3k7/Tlr4/Ntf3/Akap13/Insr	7
regulation of sensory perception	5/239	59/29008	1.27E-04	0.0235	0.0184	Tac4/Cacnb3/Il33/Tlr4/Tac1	5
detection of stimulus involved in sensory perception of pain	4/239	32/29008	1.35E-04	0.0237	0.0185	Tac4/Cacnb3/Tlr4/Tac1	4
response to BMP	8/239	184/29008	1.52E-04	0.0241	0.0189	Sfrp4/Fstl1/Smad2/Eng/Rbpj/Comp/Dand5/Cdh5	8
cellular response to BMP stimulus	8/239	184/29008	1.52E-04	0.0241	0.0189	Sfrp4/Fstl1/Smad2/Eng/Rbpj/Comp/Dand5/Cdh5	8
beta-catenin binding	6/243	94/28438	1.59E-04	0.0237	0.0195	Ptprk/Calcoco1/Cxadr/Tcf4/Ajap1/Cdh5	6
ossification	12/239	417/29008	1.95E-04	0.0295	0.0230	H3f3a/Osr1/Foxc1/Cnmd/Map3k7/Rbpj/Tmem119/Tac1/Dchs1/Comp/Wwox/Sik3	12
regulation of cellular response to growth factor stimulus	10/239	299/29008	2.03E-04	0.0295	0.0230	Sfrp4/Cnmd/Fstl1/Spry4/Tcf4/Smad2/Eng/Rbpj/Dand5/Cdh5	10
endochondral bone morphogenesis	5/239	66/29008	2.16E-04	0.0300	0.0235	Foxc1/Tmem119/Trpv4/Comp/Sik3	5
neurokinin receptor binding	2/243	3/28438	2.17E-04	0.0237	0.0195	Tac4/Tac1	2
substance K receptor binding	2/243	3/28438	2.17E-04	0.0237	0.0195	Tac4/Tac1	2
3-phosphoinositide-dependent protein kinase binding	2/243	3/28438	2.17E-04	0.0237	0.0195	Sgk1/Insr	2
saliva secretion	3/239	15/29008	2.34E-04	0.0302	0.0236	Tac4/Kcnma1/Tac1	3

regulation of chromosome condensation	2/239	5/29008	6.65E-04	0.0453	0.0354	H3f3a/Wapl	2

Table S3. GO enrichment analysis of differentially expressed downregulated genes by H3K4me3 CUT&TAG (top30)

**Description**	**GeneRatio**	**BgRatio**	**pvalue**	**p.adjust**	**qvalue**	**geneID**	**Count**
pattern specification process	19/205	482/29008	1.85E-09	0.0000	0.0000	Myf6/Hnf1b/Hoxb8/Bptf/Foxc1/Tfap2a/Barx1/Isl1/Acvrl1/Sufu/Emx2/1700007K13Rik/Acvr2a/Smarcd3/Drc1/Grsf1/Tbx3/Uncx/Tcf7l1	19
regionalization	15/205	379/29008	9.51E-08	0.0002	0.0001	Myf6/Hnf1b/Hoxb8/Bptf/Foxc1/Barx1/Isl1/Acvrl1/Sufu/Emx2/Acvr2a/Smarcd3/Grsf1/Tbx3/Tcf7l1	15
anterior/posterior pattern specification	11/205	232/29008	8.89E-07	0.0010	0.0008	Myf6/Hnf1b/Hoxb8/Bptf/Foxc1/Barx1/Emx2/Acvr2a/Grsf1/Tbx3/Tcf7l1	11
**nephric duct formation**	3/205	5/29008	3.44E-06	0.0029	0.0022	Hnf1b/Wnt9b/Gata3	3
**bone development**	10/205	243/29008	9.82E-06	0.0060	0.0046	Fgf18/Notum/Foxc1/Tfap2a/Zfp385a/Src/Trpv4/Por/Foxp1/Comp	10
cardiac right ventricle morphogenesis	4/205	20/29008	1.07E-05	0.0060	0.0046	Isl1/Gata3/Chd7/Smarcd3	4
peptidyl-serine modification	12/205	365/29008	1.24E-05	0.0060	0.0046	Akt3/Epm2a/Mgat5b/Dyrk1a/Mapk13/Camk2a/Src/Pink1/Lmtk2/Calcr/Ikbkb/Dclk3	12
regulation of epithelial cell differentiation	8/205	157/29008	1.69E-05	0.0071	0.0054	Wnt9b/Foxc1/Acvrl1/Gata3/Tbx3/Foxp1/Foxa3/Ikbkb	8
peptidyl-serine phosphorylation	11/205	340/29008	3.32E-05	0.0124	0.0095	Akt3/Epm2a/Dyrk1a/Mapk13/Camk2a/Src/Pink1/Lmtk2/Calcr/Ikbkb/Dclk3	11
**heart morphogenesis**	10/205	284/29008	3.74E-05	0.0126	0.0096	Foxc1/Tfap2a/Isl1/Acvrl1/Sufu/Gata3/Chd7/Cdc42/Smarcd3/Tbx3	10
**mesonephric duct formation**	2/205	2/29008	4.97E-05	0.0134	0.0103	Hnf1b/Wnt9b	2
**mesonephric tubule formation**	3/205	11/29008	5.50E-05	0.0134	0.0103	Hnf1b/Wnt9b/Gata3	3
**nephric duct morphogenesis**	3/205	11/29008	5.50E-05	0.0134	0.0103	Hnf1b/Wnt9b/Gata3	3
myeloid cell differentiation	12/205	426/29008	5.60E-05	0.0134	0.0103	Smap1/Hoxb8/Zfp385a/Rps14/Fech/Gata3/Acvr2a/Src/Calcr/Foxp1/Il15/Hmgb3	12
protein localization to plasma membrane	10/205	301/29008	6.09E-05	0.0134	0.0103	Epm2a/Map7/Skap1/Krt18/Camk2a/Myo5b/Pacs1/Pkp3/Ikbkb/Rdx	10
negative regulation of cell junction assembly	4/205	31/29008	6.56E-05	0.0134	0.0103	Bcas3/Acvrl1/Src/Ikbkb	4
**nephric duct development**	3/205	12/29008	7.30E-05	0.0134	0.0103	Hnf1b/Wnt9b/Gata3	3
axon guidance	9/205	255/29008	9.00E-05	0.0134	0.0103	Isl1/Gata3/Artn/Runx3/Apbb2/Lmtk2/Foxp1/Mir200c/Vasp	9
Wnt signaling pathway	12/205	449/29008	9.21E-05	0.0134	0.0103	Epm2a/Hnf1b/Wnt9b/Notum/Barx1/Isl1/Ctnnd2/Frmd8/Gnaq/Gata3/Src/Tcf7l1	12
neuron projection guidance	9/205	256/29008	9.28E-05	0.0134	0.0103	Isl1/Gata3/Artn/Runx3/Apbb2/Lmtk2/Foxp1/Mir200c/Vasp	9
cell-cell signaling by wnt	12/205	451/29008	9.61E-05	0.0134	0.0103	Epm2a/Hnf1b/Wnt9b/Notum/Barx1/Isl1/Ctnnd2/Frmd8/Gnaq/Gata3/Src/Tcf7l1	12
**mesonephric epithelium development**	6/205	104/29008	9.88E-05	0.0134	0.0103	Sim1/Hnf1b/Wnt9b/Foxc1/Gata3/Tacstd2	6
**mesonephric tubule development**	6/205	104/29008	9.88E-05	0.0134	0.0103	Sim1/Hnf1b/Wnt9b/Foxc1/Gata3/Tacstd2	6
**ureteric bud development**	6/205	104/29008	9.88E-05	0.0134	0.0103	Sim1/Hnf1b/Wnt9b/Foxc1/Gata3/Tacstd2	6
cardiac ventricle development	7/205	151/29008	1.04E-04	0.0134	0.0103	Foxc1/Isl1/Sufu/Gata3/Chd7/Smarcd3/Tbx3	7
endochondral bone morphogenesis	5/205	66/29008	1.06E-04	0.0134	0.0103	Fgf18/Foxc1/Trpv4/Por/Comp	5
skeletal system morphogenesis	9/205	261/29008	1.07E-04	0.0134	0.0103	Fgf18/Hoxb8/Wnt9b/Foxc1/Tfap2a/Trpv4/Por/Uncx/Comp	9
mesonephros development	6/205	107/29008	1.16E-04	0.0138	0.0106	Sim1/Hnf1b/Wnt9b/Foxc1/Gata3/Tacstd2	6
bone morphogenesis	6/205	108/29008	1.22E-04	0.0138	0.0106	Fgf18/Foxc1/Tfap2a/Trpv4/Por/Comp	6

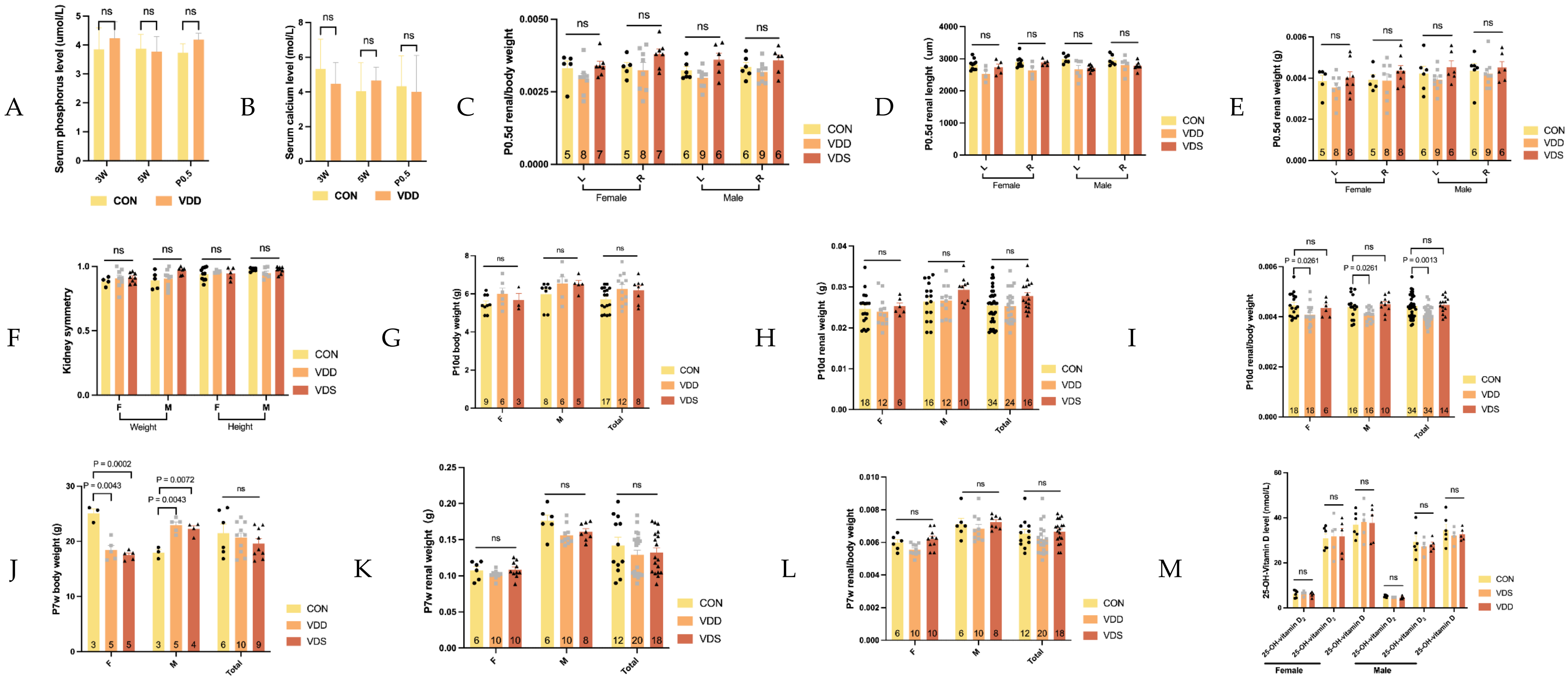

Supplementary Figure S1. Kidney changes in offspring mice with prepregnant plus pregnant vitamin D-deficient and pregnancy vitamin D-deficient. (A, B) serum calcium and phosphorus concentrations between the two groups; (C-F) Body weight, kidney weight, renal symmetry by weight at P0.5; (G-I) Body weight, kidney weight, kidney weight/body weight ratio at P10; (J-L) Body weight, kidney weight, kidney weight/body weight ratio at P7weeks. (M) vitamin D levels (nmol/L) at P7w. Each value is expressed as the mean ± SEM. VDD, pre-pregnancy plus pregnancy vitamin D-deficient group; VDS, pre-pregnancy vitamin D-deficient group; CON, pre-pregnancy plus pregnancy normal group; F: female; M: male; L: left; R: right; W: weight.

A B C D

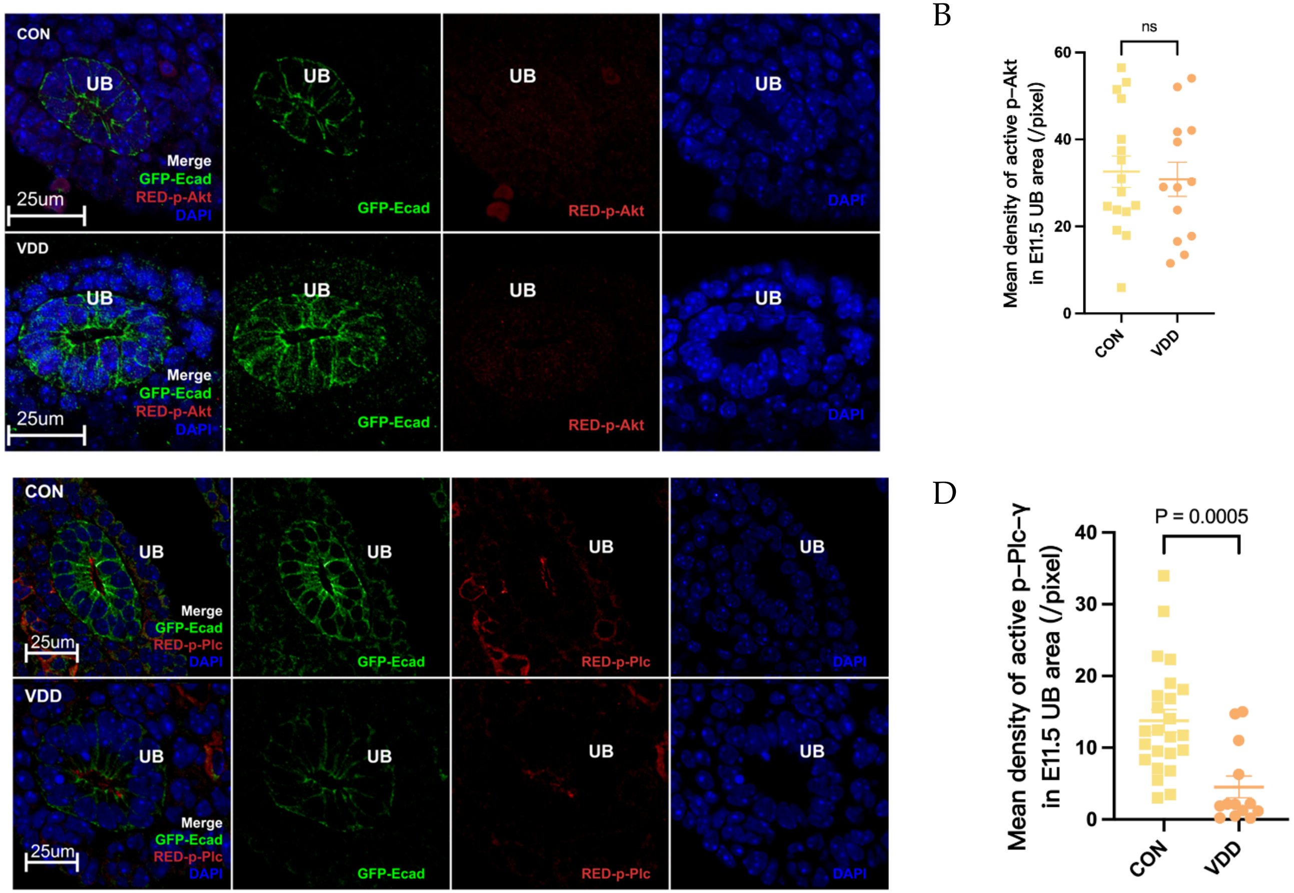

Supplementary Figure S2.

Detect the changes of p-Akt and p-Plcγ

(A, B) Detect the p-Akt expression levels of UB tissues by immunofluorescence; (C, D) Detect the p-Plcγ expression levels of UB tissues by immunofluorescence. Each value is expressed as the mean ± SEM. Abbreviations: ns, non-significant; VDD, pre-pregnancy plus pregnancy vitamin D-deficient group; VDS, pre-pregnancy vitamin D-deficient group; CON, pre-pregnancy plus pregnancy normal group; UB, Ureteric Bud; CND, common nephric duct.

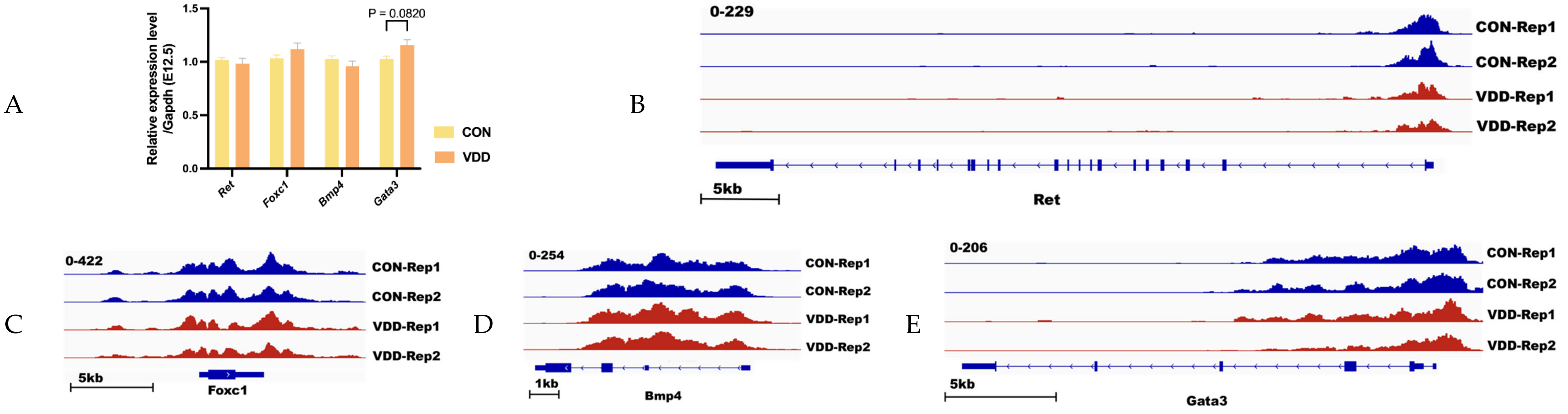

Supplementary Figure S3: H3K4me3 CUT&TAG Ret, Foxc1, Bmp4, Gata3 promoter region peak and detect the expression levels by qRT–PCR. (A) Detect the *Ret, Foxc1, Bmp4, Gata3* expression levels of E12.5 UB tissues by qRT–PCR; (B-E) H3K4me3 CUT&TAG of Ret, Foxc1, Bmp4, Gata3 promoter region peak; Each value is expressed as the mean ± SEM. Abbreviations: ns, non-significant; VDD, pre-pregnancy plus pregnancy vitamin D-deficient group; CON, pre-pregnancy plus pregnancy normal group.
